# *Ganoderma lucidum* Extract Reduces Insulin Resistance by Enhancing AMPK Activation in High-Fat Diet-Induced Obese Mice

**DOI:** 10.3390/nu12113338

**Published:** 2020-10-30

**Authors:** Hyeon A Lee, Jae-Han Cho, Qonita Afinanisa, Gi-Hong An, Jae-Gu Han, Hyo Jeung Kang, Seong Ho Choi, Hyun-A Seong

**Affiliations:** 1 Department of Biochemistry, School of Biological Sciences, Chungbuk National University, Cheongju 28644, Korea; hyeona@chungbuk.ac.kr (H.A.L.); qonita@chungbuk.ac.kr (Q.A.); 2 Mushroom Research Division, National Institute of Horticultural and Herbal Science, RDA Eumseong, Chungbuk 27709, Korea; limitcho@korea.kr (J.-H.C.); agiho@korea.kr (G.-H.A.); hanjaegu@korea.kr (J.-G.H.); 3 College of Pharmacy and Research Institute of Pharmaceutical Sciences, Kyungpook National University, Daegu 41566, Korea; 4 Department of Animal Science, Chungbuk National University, Cheongju 28644, Korea

**Keywords:** anti-obesity, *Ganoderma*, lipogenesis, AMPK, insulin resistance

## Abstract

*Ganoderma lucidum* is used widely in oriental medicine to treat obesity and metabolic diseases. Bioactive substances extracted from *G. lucidum* have been shown to ameliorate dyslipidemia, insulin resistance, and type 2 diabetes in mice via multiple 5′ AMP-activated protein kinase (AMPK)-mediated mechanisms; however, further studies are required to elucidate the anti-obesity effects of *G. lucidum* in vivo. In this study, we demonstrated that 3% *G. lucidum* extract powder (GEP) can be used to prevent obesity and insulin resistance in a mouse model. C57BL/6 mice were provided with a normal diet (ND) or a high-fat diet (HFD) supplemented with 1, 3, or 5% GEP for 12 weeks and the effect of GEP on body weight, liver, adipose tissue, adipokines, insulin and glucose tolerance (ITT and GTT), glucose uptake, glucose-metabolism related proteins, and lipogenesis related genes was examined. GEP administration was found to reduce weight gain in the liver and fat tissues of the mice. In addition, serum parameters were significantly lower in the 3% and 5% GEP mice groups than in those fed a HFD alone, whereas adiponectin levels were significantly higher. We also observed that GEP improved glucose metabolism, reduced lipid accumulation in the liver, and reduced adipocyte size. These effects may have been mediated by enhanced AMPK activation, which attenuated the transcription and translation of lipogenic genes such as fatty acid synthase (FAS), stearoyl-CoA desaturase 1 (SCD1), and sterol regulatory element-binding protein-1c (SREBP1c). Moreover, AMP-activated protein kinase (AMPK) activation increased acetyl-CoA carboxylase (ACC), insulin receptor (IR), IR substrate 1 (IRS1), and Akt protein expression and activation, as well as glucose transporter type 1/4 (GLUT1/4) protein production, thereby improving insulin sensitivity and glucose metabolism. Together, these findings demonstrate that *G. lucidum* may effectively prevent obesity and suppress obesity-induced insulin resistance via AMPK activation.

## 1. Introduction

Obesity is a metabolic disease characterized by excess lipid deposition [1,2] that can increase adipose tissue mass by increasing the number (hyperplasia) or size (hypertrophy) of adipocytes. Adipocyte hypertrophy can dysregulate adipocyte hormone signaling and increase the secretion of inflammatory cytokines, leading to insulin resistance and chronic low-grade inflammation, among other complications [3,4]. As a result of its disruptive effects on systemic metabolism, obesity is recognized as a major risk factor for multiple metabolic disorders, including type 2 diabetes, atherosclerosis, hepatic steatosis, and cancer [5,6]. In recent years, a variety of treatments have been developed to inhibit obesity. One potential therapeutic target is 5′ AMP-activated protein kinase (AMPK), an important protein kinase that responds to metabolic stress and regulates energy homeostasis. By sensing AMP/ATP depletion, AMPK regulates cellular growth and proliferation by integrating nutritional information with catabolic and anabolic processes such as glucose uptake, glycolysis, fatty acid oxidation, lipogenesis, gluconeogenesis, cholesterol production, and rRNA synthesis [7,8]. AMPK regulates lipid metabolism via multiple signaling pathways. Notably, AMPK has been reported to inhibit sterol regulatory element-binding protein 1c (SREBP1c) and peroxisome proliferator-activated receptor γ (PPARγ), which are the master regulators of lipogenesis and adipogenesis, respectively [9]. SREBP1c and PPARγ downregulation reduces the expression of lipogenic genes such as pyruvate kinase, fatty acid synthase (FAS), and acetyl-CoA carboxylase (ACC) [8,10,11]. ACC is an enzyme that converts acetyl-CoA into malonyl-CoA, a precursor of fatty acid synthesis that inhibits fatty acid oxidation in mitochondria [8]; consequently, the inhibition of ACC by AMPK reduces fatty acid synthesis and increases fatty acid oxidation [10,11]. AMPK has also been found to activate peroxisome proliferator-activated receptor gamma coactivator 1-alpha (PGC1α) and thereby stimulate mitochondrial biogenesis, leading to increased fatty acid oxidation [8]. Moreover, AMPK phosphorylation has been reported to decrease cholesterol synthesis by activating β-hydroxy β-methylglutaryl-CoA (HMG-CoA) reductase, a rate limiting enzyme in cholesterol and isoprenoid synthesis [12,13]. Consequently, as overall lipid metabolism improves, adiponectin levels return to normal, thereby increasing insulin sensitivity and reducing triglyceride levels [8]. Thus, increasing AMPK activation may reduce obesity. 

In obesity, ectopic lipid accumulation contributes toward systemic insulin resistance, which is a major predictor of the development of type 2 diabetes [2,14]. Insulin is a hormone that stimulates glucose uptake and storage and inhibits hepatic glucose production. High blood fatty acid levels induce insulin resistance by increasing the synthesis of diacylglycerol and ceramide, which inhibit insulin receptor kinase via protein kinase C (PKC) [15]. Insulin resistance describes the phenomenon when a normal or increased insulin level could not induce a proper metabolic response, such as insulin-induced glucose uptake [16]. This may result in impaired metabolism such as hyperglycemia due to insufficient blood glucose uptake, and insulin overproduction by pancreatic β cells in an attempt to increase glucose uptake [8,16]. Consequently, insulin resistance can be improved indirectly by enhancing AMPK activation to restore lipid metabolism, and directly by regulating proteins involved in glucose metabolism. Physical activity has been reported to improve glycemic control by increasing AMPK activation and upregulating glucose transport in an insulin-dependent manner [8]. AMPK activation also decreases glucose production in the liver by phosphorylating mTOR Complex 2 (TORC2) and inhibiting the gene expression of important gluconeogenic enzymes, including G6Pase and phosphoenolpyruvate carboxykinase (PEPCK) [8]. In skeletal muscles, AMPK increases the activity of Akt and its downstream targets, Akt substrate of 160 kDa /TBC1 Domain Family Member 4 (AS160/TBC1D4), which play important roles in glucose uptake by stimulating the translocation of GLUT4 to the plasma membrane [8]. Consistently, biguanides and thiazolidinediones that increase AMPK activation have been widely used as antidiabetic drugs [8]. Due to its key role in energy metabolism pathways, AMPK has become a potential target for treating obesity, insulin resistance, and type 2 diabetes [8].

Therapies for obesity and insulin resistance have included antibiotics, prebiotics, and natural compounds extracted from medicinal plants or mushrooms. Indeed, plant or mushroom extracts can contain various functional bioactive compounds that may simultaneously interact with signaling pathways involved in obesity and insulin resistance [17]. *Ganoderma lucidum,* also known as “Reishi”, “Ling Zhi”, or “Mannentake”, is a medicinal *Basidiomycetes* mushroom with potential anti-obesity effects [18]. *G. lucidum* has been used for thousands of years to improve health and longevity, and to treat diseases such as hypertension, cancer, and diabetes mellitus [17,19,20], and has been suggested as a potential agent to prevent obesity and metabolic diseases, such as type 2 diabetes, via the regulation of AMPK [9,20,21]. In addition, numerous studies have suggested that *G. lucidum* exhibits therapeutic properties, including antidiabetic, immunomodulatory, hepatoprotective, antihyperlipidemic, and antihyperglycemic activities [17,20,22,23,24,25,26]. A dietary supplement containing *G. lucidum* has been shown to inhibit adipogenesis by suppressing PPARγ, SREBP1c, and C/EBPα (CCAAT-enhancer-binding protein α), and activating AMPK to improve glucose uptake in vitro [9], while ganodermanondiol extracted from *G. lucidum* has been reported to enhance the activation of AMPK and its upstream kinases to inhibit fatty liver [27]. The polysaccharides present in *G. lucidum* have also demonstrated hypoglycemic effects mediated by AMPK activation, which enhances phosphoinositide 3-kinase (PI3K) and Akt gene expression and protein synthesis, thus increasing glucose uptake and reducing glycogen synthesis [20,28]. Furthermore, the use of herbal medicines and natural compounds has shown no apparent short nor long term side effects in the human body, making them attractive alternatives to pharmaceutical therapies [17]. *Ganoderma lucidum* extract powder (GEP) used in this study is in the form of powder which makes it practical to be produced in pill for clinical trial application. 

In this study, we verified the effect of *G. lucidum* extract on obesity in a mouse model, finding that a 3% extract exerted anti-obesity effects in vivo by reducing hyperlipidemia, recovering functional adipokine levels, upregulating glucose metabolism-related protein expression and phosphorylation, and suppressing lipogenesis-related genes. Therefore, our data suggest that 3% *G. lucidum* extract can be used as a supplement to prevent obesity and associated metabolic diseases. 

## 2. Materials and Methods 

### 2.1. G. Lucidum Extract Powder

*G. lucidum* extract powder (ASI7071; GEP) was provided by the National Institute of Horticultural and Herbal Science (Eumseong, Chungcheongbuk-do, Korea). A *G. lucidum* cultivar was obtained from the National Horticultural Research Institute and cultivated on oakwood before being desiccated using dry heat. Dried samples (0.5 g) were extracted three times with 10 mL of 95% ethanol or 20× volume per weight for 24 h. The extract was filtered under reduced pressure and concentrated by rotary evaporation using a speed-vacuum (Hanil, module 4080C, Gimpo, Gyeonggi-do, Korea). The major bioactive constituents of GEP is stated in Table 1.

### 2.2. Animals

Six-week-old specific pathogen free (SPF) C57BL/6 mice were purchased from RaonBio (Yongin, Gyeonggi-do, Korea). During the experimental period, mice were kept in a SPF environment with a controlled atmosphere (23 ± 2 °C and 55 ± 5% relative humidity) and a 12 h light/dark cycle. 

All animal experiments were approved by the Chungbuk National University, and were conducted in accordance with the approved protocols and guidelines established by the Chungbuk National University Institutional Animal Care and Use Committees (Approval No.: CBNUA-1128-18-01; approval date: 30 May 2018). After a week of acclimatization, mice were divided randomly into five groups and fed ad libitum with a normal chow diet (ND), high-fat diet (HFD), or HFD + 1, 3, or 5% GEP for 12 weeks (*n* = 6–7 mice per group). The dietary compositions used in this study are summarized in Table 2. During the 12-week experimental period, food intake was measured at 2-day intervals and body weight was measured once per week.

### 2.3. Tissue Weight Measurement

After the 12-week experimental period, the mice were fasted for 12 h and anesthetized with ether. Liver and white adipose tissues (WAT; epididymal fat) were excised, washed with phosphate buffered saline (PBS), dehydrated with filter paper, and weighed.

### 2.4. Histopathological Observation 

Epididymal WAT, peritoneal WAT, subcutaneous WAT, and some liver tissues were extracted from the mice, fixed in 10% neutral buffered formalin for 24–48 h, and rinsed with distilled water. The tissues were then dehydrated in stages using 70%, 80%, 90%, 95%, and 100% ethanol and embedded in paraffin. The paraffin block was cut into 4–5 μm thick sections, prepared into slides, washed with xylene, and hydrophilized gradually with 100%, 95%, 90%, 80% and 70% ethanol. The slides were then stained with hematoxylin and eosin, covered with a cover glass, and observed using an optical microscope (Olympus, Tokyo, Japan). 

### 2.5. Measurement of Blood Lipid Concentration

All blood analysis was performed after 12 weeks of experimental period (*n* = 6–7 per group). Blood was collected from the inferior vena cava using a sterile syringe before being allowed to stand in ice for 1 h and centrifuged at 3000 rpm (4 °C) for 10–15 min. The supernatant serum was collected and triglyceride (TG), total cholesterol (TC), high-density lipoprotein-cholesterol (HDL), low-density lipoprotein-cholesterol (LDL), and free fatty acid (FFA) levels were measured using an automated biochemical analyzer (Hitachi 7080; Hitachi Science System Ltd., Chiyoda-ku, Tokyo, Japan).

### 2.6. Measurement of Blood Glucose Concentration

After the 12-week experimental period and 12 h of fasting, blood samples were collected from the tail and glucose was measured using a blood glucose meter (Accu-Check glucometer, Roche, Seoul, Gyeonggi-do, Korea).

### 2.7. Glucose and Insulin Tolerance Tests (GTTs and ITTs)

A separate set of mice (*n* = 6–7 per group) was used for ITT and GTT measurements after 12 weeks of treatment. HFD-induced obese mice were fasted for 18 h for GTT or 6 h for ITT and intraperitoneally injected with D-glucose (2 g/kg body weight) or insulin (0.75 U/kg body weight in 0.1% bovine serum albumin) [29]. Blood samples were taken from the tail and blood glucose levels were measured using an Accu-Check glucometer (Roche) at the indicated times.

### 2.8. Measurement of Insulin, Leptin, and Adiponectin Levels

Blood insulin, leptin, and adiponectin concentrations were measured using an ELISA reader with corresponding mouse ELISA kits (CrystalChem, Inc., Elk Grove Village, IL, USA). Blood was collected from the inferior vena cava with a sterile syringe after the abdomen had been opened to analyze blood indices. The collected blood was allowed to stand for 1 h on ice and centrifuged at 3000 rpm (4 °C) for 10–15 min. The serum supernatant was diluted 10 times and a 50 μL aliquot was reacted at 4 °C for 12 h before incubation with HRP-anti-mouse insulin, leptin, and adiponectin antibodies at 4 °C for 4 h. Non-specifically bound antibodies were removed by washing with phosphate buffered saline-tween (PBS/T) twice. After treatment with substrate solution for 30 min, absorbance was measured at 450 and 630 nm using the Benchmark Plus microplate reader (BioRad, Hercules, CA, USA).

### 2.9. Measurement of 2-Deoxy-Glucose Uptake Following In Vivo Insulin Stimulation

After the 12-week experimental period, mice were fasted for 16 h, anesthetized with sodium pentobarbital (50 mg/kg; P3761, Sigma), and sacrificed. Epididymal WATs were harvested, preincubated in KBH buffer (8 mM glucose, 5 mM HEPES, 0.1% bovine serum albumin (fraction V), and 2 mM sodium acetate) at 30 °C for 20 min, and then incubated in KBH buffer containing 0.3 μCi [^14^C] mannitol and 2 μCi/mL 2-[^3^H]deoxy-glucose without 100 nM human insulin in a shaking water bath at 30 °C for 30 min. The tissues were blotted, rapidly freeze-clamped, and weighed before being dissolved in 0.5 mL of 1 M KOH for 20 min at 70 °C and neutralized with 0.5 mL of 1 M HCl. A 0.3 mL aliquot of each sample was mixed with 6 mL of Biosafe II (Research Products International, Mount Prospect, IL, USA) and radioactivity quantified using a liquid scintillation counter. The [^14^C] mannitol concentration was measured to quantify extracellular 2-[^3^H] deoxy-glucose and glucose uptake was calculated by subtracting extracellular 2-[^3^H] deoxy-glucose from total 2-[^3^H] deoxy-glucose.

### 2.10. qRT-PCR of Lipogenesis Genes in Adipose and Liver Tissues

Quantitative reverse transcription-PCR (qRT-PCR) was performed to identify changes in the expression of obesity-related genes in adipose and liver tissues. Total RNA was extracted from rapidly-frozen adipose and liver tissues using a RNeasy Lipid Tissue Mini Kit and a RNeasy Mini Kit (Qiagen, Germantown, MD, USA) according to the manufacturer’s protocols. The extracted RNA was reverse transcribed using a SuperScript^®^ IV First-Strand Synthesis System (Invitrogen, Waltham, MA, USA) and the expression of lipogenesis-related genes was measured using real-time PCR with Fast SYBR Green Master Mix (Applied Biosystems, Waltham, MA, USA), 25 μL of real-time PCR reaction mix, 2 μL of cDNA, 12.5 μL of 2X SYBR mix, and 1 μL of forward and reverse primers (Table 3; 10 pmol/μL). PCR amplification was carried out for 40 cycles of pre-denaturation (95 °C) for 15 min, denaturation (95 °C) for 15 s, annealing (55 °C) for 30 s, and extension (72 °C) for 30 s. The expression of 18s ribosomal RNA was used as an internal control to correct relative gene expression. Melting curve analysis was used to confirm primer specificity. Results were analyzed using One-step system software v2.1 (Applied Biosystems).

### 2.11. Quantification of Glucose and Lipid Metabolism-Related Protein Expression in WATs

WATs were harvested from male C57BL/6 mice in each group, treated with insulin, solubilized with NP-40 lysis buffer (20 mM HEPES, pH 7.9, 10 mM EDTA, 0.1M KCl, 0.3 NaCl, 10 µg/mL aprotinin, 10 µg/mL leupeptin, 2 µg/mL alpha1-antitrypsin, 2 mM sodium pyrophosphate, 1 mM sodium orthovanadate, 10 mM sodium fluoride, 1 mM phenylmethylsulfonyl fluoride (PMSF), 25 mM sodium b glycerophosphate, and 0.1% Nonidet P-40), and pre-cleared with Protein A-Sepharose CL-4B (GE17-0780-01; GE Healthcare) for 1 h. Immunoprecipitants or whole-cell extracts were washed with lysis buffer and analyzed using sodium dodecyl sulphate-polyacrylamide gel electrophoresis (SDS-PAGE), and western blot. Protein bands were transferred from SDS gels to PVDF membrane using TE22 Mighty Small Transphor Unit (Hoefer, CA, USA), and detected using corresponding primary antibodies diluted to 0.2 µg/ml. Anti-phospho-IRβ(Y1322) (#04-300) and anti-IRβ (MABN390) antibodies were from Millipore Corp (MA, USA). Anti-SREBP-1 (sc-13551), anti-FAS (sc-74540), anti-phospho-IRS1(Y989) (sc-17200), and anti-IRS1 (sc-8038) were from Santa Cruz Biotechnology (TX, USA). Anti-phospho-AMPKα(Thr172) (#2531), anti-AMPKα (#5832), anti-phospho-ACC1(Ser79) (#11818), anti-ACC1 (#4190), anti-phospho-AKT1 (T308) (#2965), anti-phospho-AKT1 (S473) (#9018), anti-AKT1 (#2938), and anti-SCD1 (#2438) were from Cell Signaling Technology (MA, USA). Anti-GLUT1 (ab652), and anti-GLUT4 (ab654) antibodies were from Abcam (Cambridge, UK). Anti-β-actin antibody (A2228) was from Sigma (MO, USA). β-actin wa used as control [30,31]. Immunoreactive signals were detected using an EzWestLumi plus kit (WSE-7120L, Atta, Japan) with X-ray films, and band densitometry was quantified with Image J software (National Institutes of Health, Bethesda, MD, USA).

### 2.12. Statistical Analysis

Data are expressed as the mean ± standard deviation and represent at least three independent experiments. Statistical significance was determined by one-way analysis of variance (ANOVA), followed by Tukey’s multiple comparison test using GraphPad Prism software (GraphPad Software, San Diego, CA, USA).

## 3. Results

### 3.1. Effect of G. Lucidum Extract on Obesity

**Figure 1:**
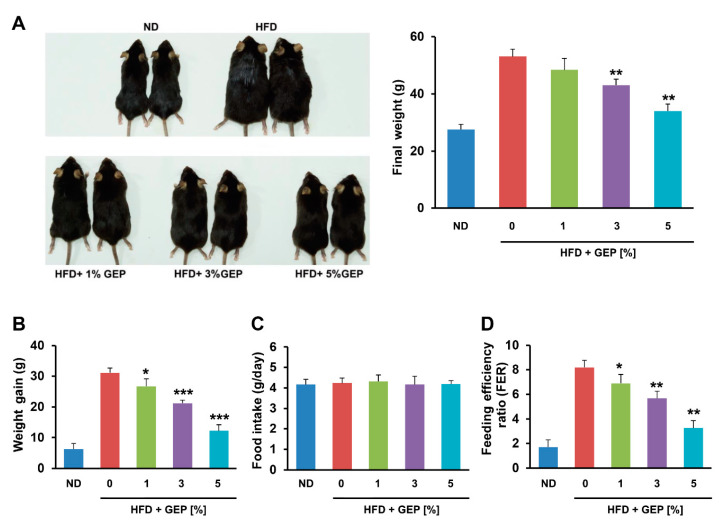
*G. lucidum* extract powder (GEP) treatment for 12 weeks reduces weight gain and the feeding efficiency ratio (FER) in HFD-induced obese mice without altering food intake. (**A**) Mouse appearance and final weight, (**B**) weight gain, (**C**) food intake, and (**D**) FER. Data represent the mean ± SD of six to seven individual mice. Significant differences vs. the HFD group (control) were determined by one-way ANOVA; * *p* < 0.1, ** *p* < 0.01, *** *p* < 0.001. ND: normal chow diet group; HFD: high-fat diet group; HFD + GEP: high-fat diet and *Ganoderma lucidum* extract powder.

To investigate the effects of *G. lucidum* on obesity, we measured the development of obesity in HFD-induced mice treated with GEP for 12 weeks. As expected, the HFD increased the size and final weight of the mice; however, GEP slightly reduced their size and significantly restored final weight (Figure 1A). In particular, significant weight gain was observed in the HFD group that was suppressed in dose-dependent manner by GEP treatment (Figure 1B). Neither the HFD nor GEP affected food intake, indicating that the effect of GEP on HFD-induced obesity may be attributed to metabolic alterations (Figure 1C). In addition, the feeding efficiency ratio (FER) suggested that GEP treatment reduced the body mass gained per calorie eaten (Figure 1D), whereas subcutaneous, epidydimal, and mesenteric WAT weight gain followed trends similar to those observed for body weight (Table 4).

**Figure 2:**
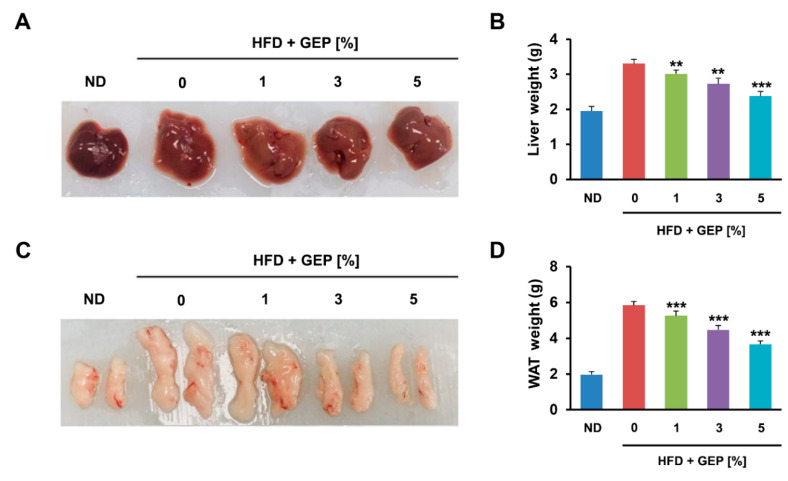
GEP decreases the size and weight of liver and WAT. (**A**) Liver size, (**B**) liver weight, (**C**) epididymal WAT size, and (**D**) total WAT weight measured using a microbalance. Data represent the mean ± SD of six to seven individual mice. Significant differences vs. the HFD group (control) were determined by one-way ANOVA; ** *p* < 0.01, *** *p* < 0.001. ND: normal chow diet group; HFD: high-fat diet group; HFD + GEP: high-fat diet and *Ganoderma lucidum* extract powder.

We also found that 12 weeks of the HFD increased the size of the liver and epididymal WAT compared to the ND (Figure 2A,C) and significantly increased liver and total WAT weight; however, GEP treatment reduced weight in a dose-dependent manner (Figure 2B,D). These results indicate that GEP can reduce weight gain, which is an obesity parameter.

### 3.2. Effect of G. Lucidum Extract on the Liver and Adipose Tissue

**Figure 3:**
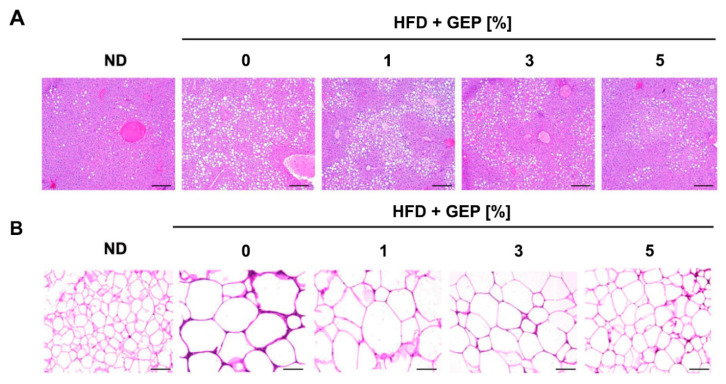
Histological analysis of (**A**) hepatocytes and (**B**) WAT sections using hematoxylin and eosin staining. Fat was lost during processing; white indicates fat. ND: normal chow diet group; HFD: high-fat diet group; HFD + GEP: high-fat diet and *Ganoderma lucidum* extract powder.

Liver and epidydimal WAT were stained to determine the effect of GEP on each tissue. The HFD group displayed greater fat accumulation than the control group, while GEP supplementation, particularly at 3% and 5%, attenuated lipid accumulation in the liver and reduced the size of adipocytes in epidydimal WAT (Figure 3A,B). Thus, GEP may reduce intracellular fat accumulation in both adipocytes and hepatocytes, which positively correlates with reduced obesity. 

### 3.3. Effect of G. Lucidum on Serum Lipid Levels

Serum samples were bioanalyzed to determine the effect of GEP on serum TG, TC, HDL, LDL, and FFA levels. Compared to the ND, the HFD significantly increased all serum obesity parameters analyzed, whereas GEP treatment lowered TG, TC, HDL, LDL, and FFA levels (Table 5). In the ND group, serum analysis detected TG levels of 73.78 ± 1.5 mg/dL, TC levels of 110.71 ± 2.79 mg/dL, HDL levels of 97.29 ± 3.83 mg/dL, LDL levels of 35.92 ± 2.96 mg/dL, and FFA levels of 0.98 ± 0.04 mEq/dL (Table 5). The HFD increased TG levels to 123.04 ± 3.12, whereas treatment with 1, 3, or 5% GEP reduced TG levels to 118.91 ± 3.04, 104.09 ± 3.66, and 88.83 ± 1.83 mg/dL, respectively. Overall, 1% GEP slightly reduced serum lipid levels while 3% and 5% GEP reduced the levels significantly. A similar pattern was observed for TC, HDL, LDL, and FFA levels, suggesting that GEP alleviates increased cholesterol and lipid concentrations in the blood.

### 3.4. Effect of G. Lucidum Extract on Glucose Uptake, Insulin Tolerance, and Glucose Tolerance

**Figure 4:**
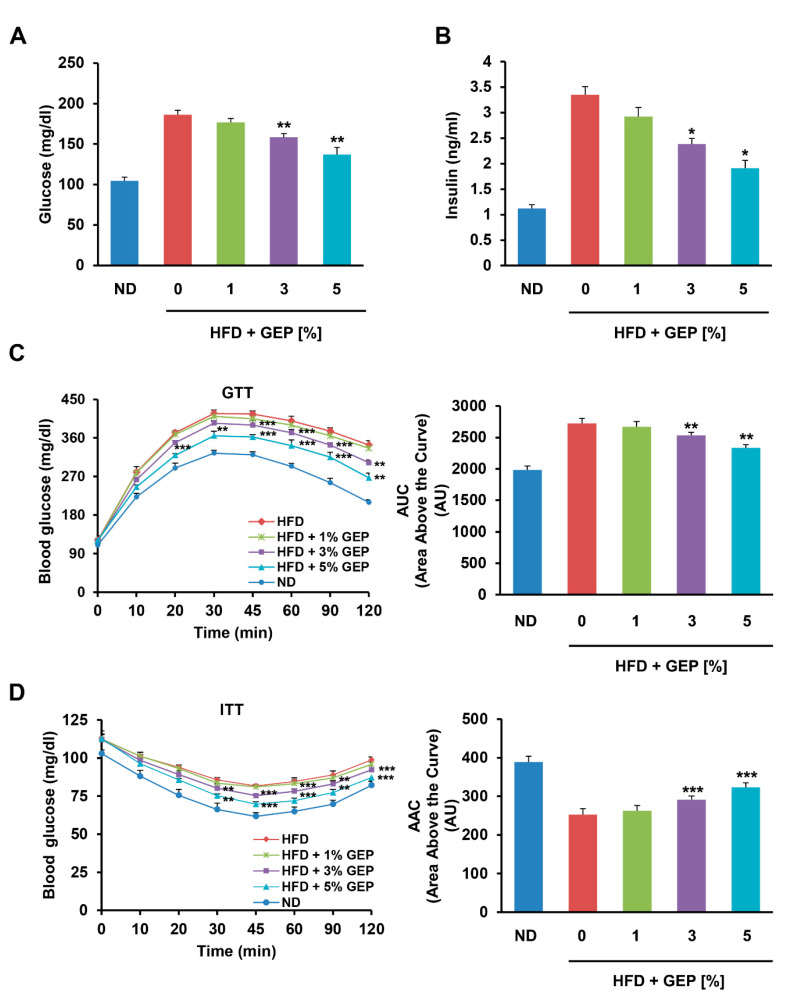
GEP improved glucose metabolism. Serum (**A**) glucose and (**B**) insulin levels after 12 weeks of GEP treatment. (**C**) Glucose tolerance test (GTT) and the area above the curve (AAC). (**D**) Insulin tolerance test (ITT) and the AUC. Data represent the mean ± SD of six to seven individual mice. Significant differences compared to the HFD group (control) were determined by one-way ANOVA; * *p* < 0.1, ** *p* < 0.01, *** *p* < 0.001. ND: normal chow diet group; HFD: high-fat diet group; HFD + GEP: high-fat diet and *Ganoderma lucidum* extract powder.

Next, we examined the effects of the HFD and GEP on glucose and insulin. After 12 weeks of treatment, the HFD increased serum glucose and insulin levels compared to the ND, while treatment with 1, 3, and 5% GEP for 12 weeks significantly reduced glucose and insulin levels in a dose-dependent manner, with a more pronounced effect on insulin levels (Figure 4A,B).

### 3.5. Effect of G. Lucidum on Adiponectin and Leptin Levels

We also performed GTTs and ITTs to measure the ability of mice to retain circulatory glucose levels over time after glucose and insulin administration (Figure 4C,D) [32]. In addition, we produced glycemia profiles from the GTT and ITT results using area above the curve (AAC) and area under the curve (AUC) analyses. We found that glucose tolerance, insulin sensitivity, as well as AAC and AUC glycemia profiles were impaired in HFD-induced obese mice compared to the ND group; however, treatment with 3% and 5% GEP significantly improved glucose tolerance and insulin sensitivity in a dose-dependent manner. Similar trends were observed for the GTT AAC and ITT AUC, with the HFD significantly increasing the AAC and reducing the AUC, but GEP treatment gradually restoring these profiles to normal levels as the GEP concentration increased. This suggests that GEP improves insulin regulation and glucose metabolism.

**Figure 5:**
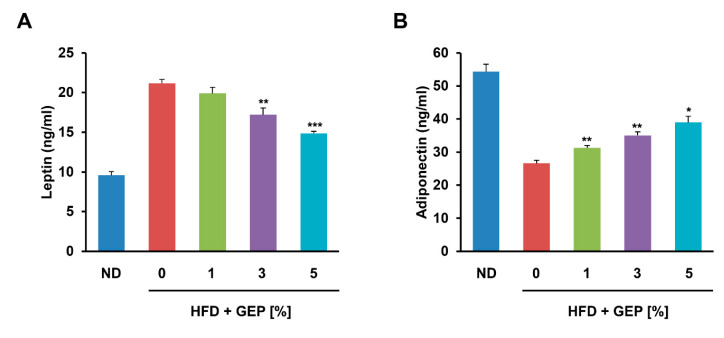
GEP treatment restored the regulation of the adipokines (**A**) leptin and (**B**) adiponectin. Data represent the mean ± SD of six to seven individual mice. Significant differences vs. the HFD group (control) were determined by one-way ANOVA; * *p* < 0.1, ** *p* < 0.01, *** *p* < 0.001. ND: normal chow diet group; HFD: high-fat diet group; HFD + GEP: high-fat diet and *Ganoderma lucidum* extract powder.

Next, we examined the effect of GEP on leptin and adiponectin in HFD-induced obese mice, observing that their production correlated positively and negatively with adiposity, respectively. GEP significantly increased and decreased leptin and adiponectin levels in a dose-dependent manner, respectively (Figure 5A,B), indicating that GEP may ameliorate adipokine regulation in obese mice.

### 3.6. Effect of G. Lucidum on Glucose Uptake and Glucose Metabolism-Related Proteins

**Figure 6:**
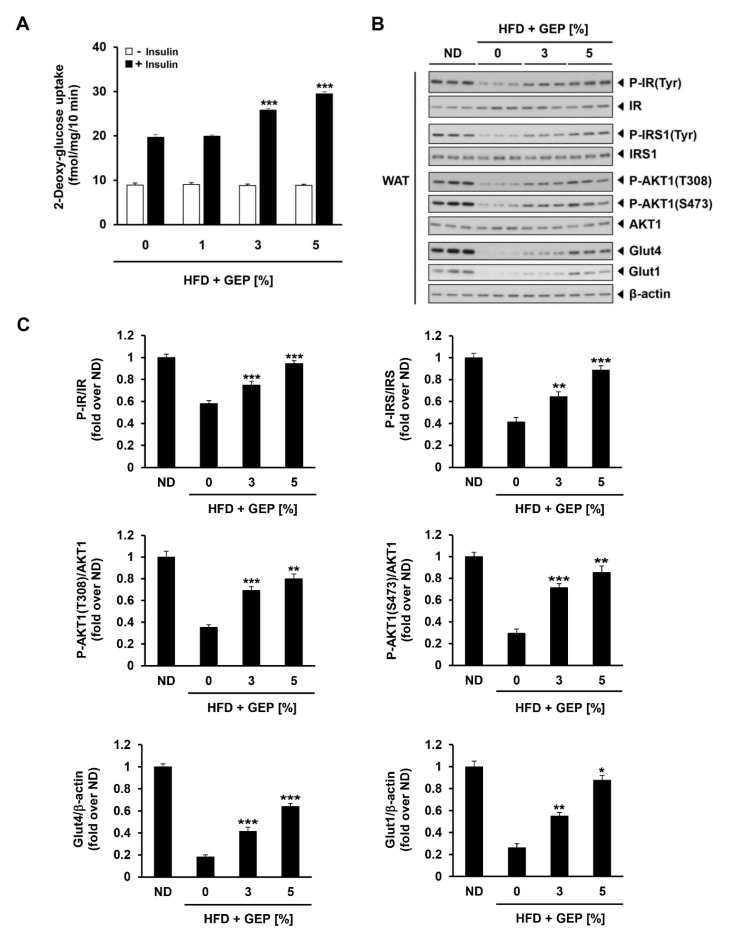
GEP effect on glucose uptake and insulin signaling. (**A**) In vivo 2-deoxy-glucose uptake in WAT treated with and without human insulin (10 mU/mL). (**B**) Western blot analysis of proteins related to insulin signaling in the presence of human insulin (10 mU/mL). (**C**) The phosphorylation ratio of IR, IRS, and AKT1, and the expression of Glut4 and Glut1 compared to β-actin, presented in folds over ND. Data represent the mean ± SD of six to seven individual mice. Significant differences vs. the HFD group (control) were determined by one-way ANOVA; * *p* < 0.1, ** *p* < 0.01, *** *p* < 0.001. HFD: high-fat diet group; GEP: *Ganoderma lucidum* extract powder. β-actin was used as a relative density control.

We also investigated GEP effect on glucose metabolism by assessing glucose uptake in the presence and absence of human insulin in WAT from 1%, 3% and 5% GEP treated HFD-induced obese mice, and the expression and activation of proteins involved in insulin signaling. In HFD-induced obese mice administered with insulin, GEP treatment dose-dependently improved glucose uptake (Figure 6A) and insulin receptor (IR), IR substrate 1 (IRS1), and AKT serine/threonine kinase 1 (AKT1) phosphorylation, thereby increasing glucose transporter type 4 and 1 (GLUT4 and GLUT1) levels as well as AKT1 activation at phosphorylation sites T308 and S473 (Figure 6B,C). These findings indicate that GEP supplementation may improve glucose metabolism. 

### 3.7. Effect of G. Lucidum Extract on Lipogenesis-Related Genes and Proteins in WAT and the Liver

**Figure 7:**
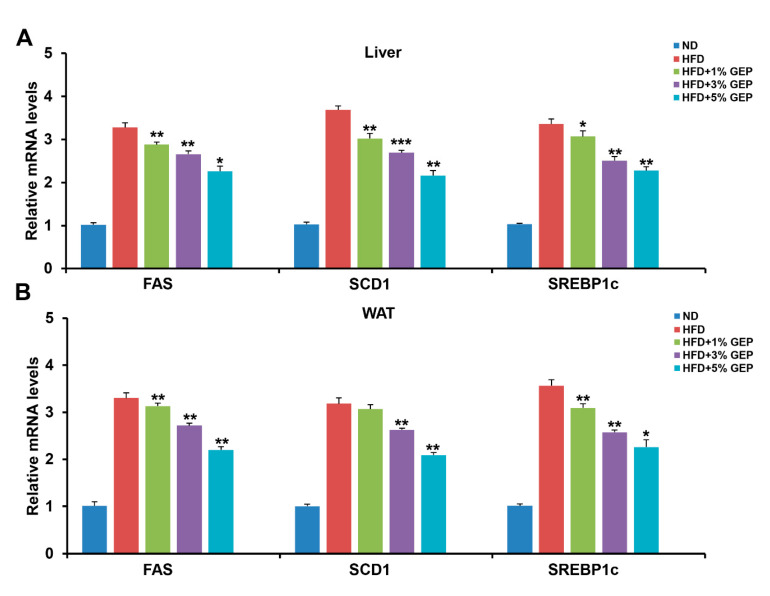
GEP treatment improved the mRNA expression of the lipogenesis-related genes FAS, SCD1, and SREBP1c. mRNA expression of lipogenesis-related genes in (**A**) the liver and (**B**) WAT measured using qRT-PCR. The mRNA levels were normalized to 18s rRNA. Data represent the mean ± SD of six to seven individual mice. Significant differences vs. the HFD group (control) were determined by one-way ANOVA; * *p* < 0.1, ** *p* < 0.01, *** *p* < 0.001. ND: normal chow diet group; HFD: high-fat diet group; HFD + GEP: high-fat diet and *Ganoderma lucidum* extract powder.

To confirm the effect of GEP on lipogenesis at the molecular level, we conducted genetic and protein analyses on WAT and the liver and analyzed fatty acid synthase (FAS), stearoyl-CoA desaturase 1 (SCD1), and sterol regulatory element-binding protein-1c (SREBP1c) expression using qRT-PCR. We found that the HFD increased the expression of lipogenesis-related genes (FAS, SCD1, SREBP1c) in both tissues compared to the ND, while 3% and 5% GEP attenuated the expression of all genes tested in a dose-dependent manner (Figure 7A,B). Together, these results confirm that GEP downregulates the mRNA expression of lipogenesis-related genes, thus inhibiting fat formation. 

**Figure 8:**
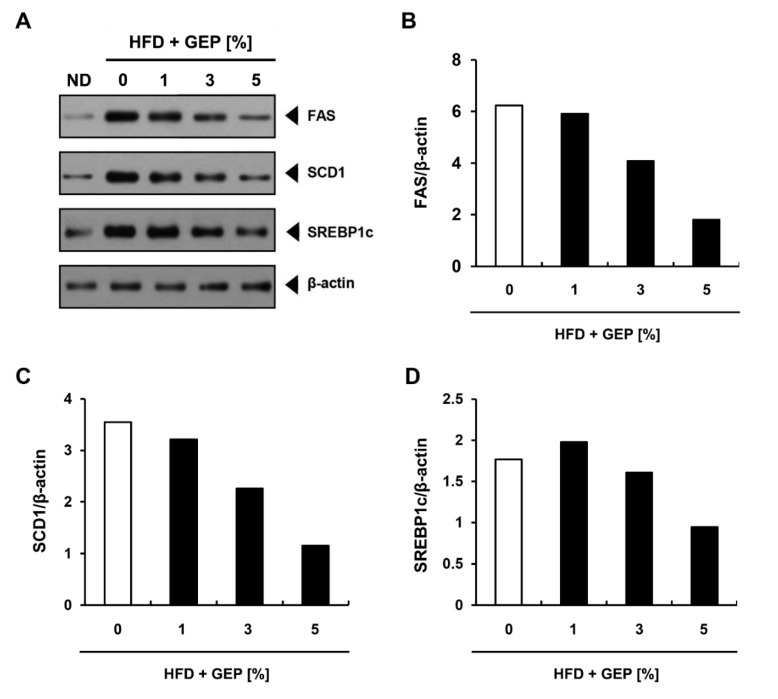
Effect of GEP on lipogenesis-related protein expression. (**A**) Western blot analysis and the expression of (**B**) FAS, (**C**) SCD1, and (**D**) SREBP1c relative to β-actin. ND: normal chow diet group (control); HFD: high-fat diet group; HFD + GEP: high-fat diet and *Ganoderma lucidum* extract powder.

To investigate the effect of GEP on lipogenic protein expression, we performed western blotting using β-actin as the control (Figure 8A). Although HFD increased the ratio of lipogenesis-related proteins (FAS, SCD 1, SREBP1c) compared to β-actin levels, 3% and 5% GEP decreased this ratio (Figure 8B–D), suggesting that GEP inhibits lipogenesis and thus obesity.

### 3.8. Effect of G. Lucidum Extract on AMPK and ACC

**Figure 9:**
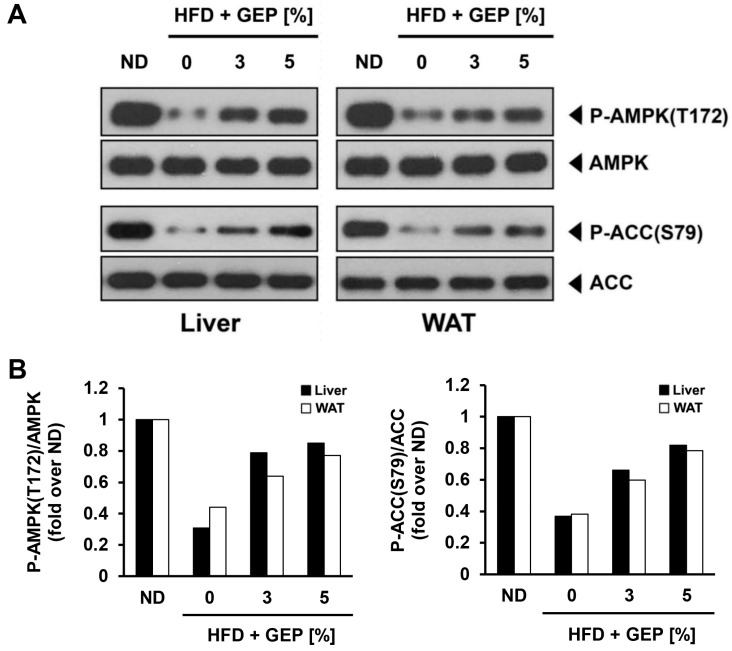
Effect of GEP on AMPK and ACC protein expression and activation. (**A**) Western blot analysis and the phosphorylation ratio of (**B**) AMPK and ACC in the liver and WAT of HFD-induced obese mice stated in fold over ND.

GEP supplementation in HFD-induced obese mice enhanced AMPK phosphorylation, leading to the activation of its downstream targets such as ACC (Figure 9A,B) in a dose-dependent manner; however, GEP did not affect the expression of AMPK or ACC. 

## 4. Discussion

*G. lucidum* has been used to treat metabolic diseases and obesity in oriental medicine; however, there is a lack of biochemical evidence to support its reported anti-obesity activities. In this study, we demonstrated the anti-obesity effects of GEP in an HFD-induced obese C57BL/6 mouse model. The mice were administered varying concentrations of GEP for 12 weeks and then body weight gain, liver and WAT weight, blood parameters, histological features, and the expression of lipogenic genes and proteins were determined. 

We observed insignificant improvements in the tested obesity parameters in mice fed a HFD + 1% GEP, while the HFD + 3% and 5% GEP groups displayed significantly lower weight gain and liver and fat tissue weights than the HFD group. Despite no change in food intake, the HFD + 3% and 5% GEP decreased the FER, suggesting that the observed weight loss may be partly attributed to improved energy metabolism. A previous study proposed that GEP concentrations above 4% can effectively improve obesity from the perspective of gut microbiota [33]. Consistently, our study suggests that the consumption of >3% GEP may improve obesity.

Obesity is characterized by adipocyte dysfunction in which lipids can no longer be stored intracellularly and spill over into the blood, causing dyslipidemia [3]. Obesity-induced dyslipidemia is marked by increased TG and LDL, and decreased HDL levels [34]; however, the HFD-induced obese mice treated with GEP displayed lower serum TG, TC, HDL, LDL, and FFA levels, suggesting that GEP may effectively prevent and alleviate hyperlipidemia and cardiovascular diseases induced by obesity. In addition, histological analysis of the liver and WAT revealed that GEP can reduce intracellular fat accumulation in both adipocytes and hepatocytes, which appears to improve obesity. Together, these results indicate that GEP reduces body weight by reducing adipocyte hypertrophy. 

Obesity is a major risk factor for metabolic diseases such as type 2 diabetes [3]. In this study, we measured serum glucose and insulin levels, insulin and glucose tolerance, and glycemia AAC and AUC as characteristics of type 2 diabetes. We demonstrated that GEP can lower serum glucose and insulin levels and improve glucose and insulin tolerance in HFD-induced obese mice, suggesting that GEP reduced insulin resistance and improved glucose metabolism in the body. In addition, we demonstrated that GEP improved glucose metabolism by enhancing the phosphorylation of IR, IRS, and Akt, thereby increasing GLUT4 and GLUT1 levels. Akt is a downstream effector in the insulin signaling pathway that, alongside AMPK, inhibits AS160 to downregulate GLUT4 translocation to the plasma membrane [35]. Thus, the activation of IR, IRS, and Akt may increase GLUT4 levels and glucose transport.

The anti-obesity effects of GEP were confirmed by analyzing the mRNA and protein expression of lipogenesis-related genes, revealing that GEP decreased FAS, SCD1, and SREBP1c mRNA and protein expression. SREBP1c is an adipogenic transcription factor that regulates several key genes in adipogenesis such as PPARγ, C/EBPα, FAS, and SCD1 [36]. In addition, SREBP1c mediates the regulation of leptin and adiponectin expression, thus plays an important role in controlling adipogenesis, lipid metabolism, and insulin sensitivity [37]. FAS is a central enzyme in de novo lipogenesis that catalyzes the conversion of malonyl-CoA into palmitate [36], while SCD1 is an enzyme involved in unsaturated fatty acid production that forms a complex with NADH-cytochrome b5 reductase and cytochrome b5 to convert stearoyl-CoA into oleoyl-CoA [38]. The anti-obesity effects of GEP could be attributed to its suppression of these lipogenic genes; however, 1% GEP appeared to increase SREBP1c levels but decreased FAS and SCD1 levels, suggesting that the lipogenesis inhibiting activities of GEP may also be linked to other regulatory mechanisms.

Adipocyte hypertrophy causes dysregulated adipokine secretion that may in turn disrupt metabolism and homeostasis [3]. Leptin is an anorexigenic adipokine that controls food intake by inhibiting appetite. In normal individuals, insulin upregulates leptin production to increase satiety when glucose levels are high; however, obesity induces leptin resistance, resulting in high blood leptin concentrations and high food intake [39]. Adiponectin is an adipokine that stimulates insulin sensitivity, glucose uptake, and fatty acid oxidation, while preventing fatty acid influx into the liver [40]. Consequently, increased adiponectin levels correlate positively with obesity improvements. The effects of both leptin and adiponectin in obesity may be mediated by AMPK since their receptor binding can activate AMPK, which inactivates acetyl-CoA carboxylase (ACC1) [41]. The inactivation of ACC1 reduces the production of malonyl-CoA, a carnitine acyl transferase (CPT1) inhibitor that is important for fatty acid β-oxidation [30]. Thus, increased AMPK activation can enhance β-oxidation and decrease the expression of SREBP1c, a transcription factor involved in lipogenesis and cholesterol synthesis. Adiponectin may also suppress some of the effects of lipid accumulation by activating ceramidase which breaks down ceramide, a molecule that stimulates fatty acid import, leads to lipid accumulation, and disrupts insulin activity. Overall, leptin and adiponectin increase lipid oxidation and decrease fatty acid synthesis and glucose output, thereby enhancing insulin sensitivity [42,43]. In this study, we demonstrated that GEP can reduce leptin and increase adiponectin levels in a dose-dependent manner. GEP restored leptin function, thus reducing hyperleptinemia, and increased adiponectin function to improve lipid and glucose metabolism. These results were consistent with the GEP antiobesity effect observed in other experiments.

Our findings also revealed that treatment with 3% or 5% GEP significantly improved obesity and insulin resistance parameters, as well as the expression and activation of proteins related to insulin signaling pathways. To investigate this further at the molecular level, we assessed AMPK and ACC expression and phosphorylation levels in the liver and WAT of mice treated with 3% or 5% GEP. Notably, GEP enhanced AMPK and ACC activation in a dose-dependent manner but independently of initial protein levels, confirming that the antihyperglycemic and antihyperinsulinemic activities of GEP might be mediated by its ability to increase AMPK phosphorylation [9,27]. Since AMPK can be activated pharmacologically to improve glucose and lipid metabolism in insulin-resistant mice, it could be a promising new target for treating type 2 diabetes [8]. The binding of AMP to the AMPK γ subunit induces conformational changes that allow upstream kinases to phosphorylate AMPK at Thr172 in its α subunit [44]. Activated AMPK can then relay signals to induce catabolism and suppress anabolism, thereby ameliorating obesity and insulin resistance [8,44]. The role of AMPK in metabolic regulation is mediated by various downstream effectors, including ACC, FAS, and GLUT4. ACC converts acetyl-CoA to malonyl-CoA, a fatty acid precursor that downregulates fatty acid oxidation by inhibiting CPT1 [45], thus increasing the availability of FFAs to synthesize other metabolites such as diacylglycerol (DAG) and ceramide [46]. DAG activates PKC which phosphorylates and inhibits insulin receptor kinase (IRK), leading to insulin resistance, while ceramide can inhibit glucose uptake by downregulating GLUT4 at the transcriptional level and inhibiting its localization to the membrane [15,35,47]. Therefore, ACC phosphorylation and inhibition by AMPK may improve GLUT4 localization and insulin sensitivity. A previous study reported that the F31 polysaccharide of *G. lucidum* might activate AMPK to suppress enzymes that regulate glucose metabolism and inhibit glucose production in the liver, thereby increasing GLUT4 expression and improving insulin resistance [20]. AMPK activation has also been shown to decrease WAT, liver, and body weight by suppressing the expression of adipogenic genes such as FAS and ACC1 [20]. Moreover, β-heteropolysaccharides extracted from *G. lucidum* have been shown to reduce fasting serum glucose and insulin levels, downregulate the expression of hepatic glucose regulatory enzyme genes, enhance AMPK activation, and increase GLUT4 mRNA and protein levels [20]. F31 polysaccharides and β-heteropolysaccharides contained in *G. lucidum* may be some of the active substances that exerted the anti-obesity effects via AMPK activation.

The regulation of insulin sensitivity by AMPK might also be linked to the mTOR pathway, which deregulates insulin signaling by activating S6K1, which phosphorylates IRS at multiple serine residues to inhibit its action and reduce glucose uptake [48,49]. In particular, AMPK inhibits mTOR/S6K1 activation, thereby enhancing IR and IRS activation and improving insulin sensitivity [49]. Indeed, studies have shown that *G. lucidum* may also exert antidiabetic effects by suppressing PEPCK gene expression and inhibiting PTP1B activity, leading to decreased blood glucose levels and improved glucose metabolism [13,50]. 

GEP used in this study contains myriads of ganoderic acids and glucans (Table 1). Ganoderic acid A has been demonstrated to reduce obesity via SREBP which is a downstream effector of AMPK [51]. Beta 1,3/1,6 glucan serves as immunomodulator in high-cholesterol induced inflammation [52]. Inflammation is one of the factors that contributes to insulin resistance [5]. *G. lucidum*’s glucans ability to alleviate inflammation might help reducing insulin resistance. 

Consistent with other works, the findings of this study suggest that *G. lucidum* extract reduces obesity by targeting AMPK. By activating AMPK, the master regulator of energy homeostasis [46], *G. lucidum* not only ameliorated obesity, but also improved overall systemic metabolism, including peripheral insulin sensitivity. This study used crude *G. lucidum* extract which is easier to procure and more practical for clinical application. We also showed that the use of crude *G. lucidum* extract does not cause adverse effects to C57BL/6 mice. A previous study by Chang, et al. [21] showed *G. lucidum* ability to significantly reduce obesity using 4% of the extract. Our findings demonstrated that 3% can have significant effects to reduce obesity and insulin resistance, thus minimizing the concentration needed for clinical therapy. Future studies should investigate the anti-obesity effects of each active substances purified from *G. lucidum*, and GEP application in clinical trial.

## 5. Conclusions

This study revealed that *G. lucidum* could effectively prevent obesity and insulin resistance by attenuating adipocyte hypertrophy and dyslipidemia and improving homeostasis by recovering insulin sensitivity and adipokine function in HFD-induced obese C57BL/6 mice. In particular, *G. lucidum* exhibited anti-obesity activities by downregulating FAS, SCD1, and SREBP1c, and also displayed antihyperglycemic and antihyperinsulinemic activities by enhancing the activation of AMPK, ACC, IR, IRS, and Akt. Together, these results suggest that *G. lucidum* extract has the potential to be used as a nutritional supplement to prevent and ameliorate obesity and type 2 diabetes. Future studies should assess the effects of *G. lucidum* bioactive compounds on other pathways related to obesity in vivo.

## Figures and Tables

**Table 1:** The major bioactive substances in *G. lucidum*.

Compounds	Contents (%)
Ganoderic Acid	
A	2.74
B	3.68
C1	0.69
C2	0.17
C6	10.39
D	0.95
F	1.09
G	2.93
H	6.98
I	1.1
K	2.3
L	0.57
LM2	5.43
M	12
N	1.15
T-Q	1.1
Total ganoderic acid	53.27
Glucan	
α-glucan	3.40 ± 0.19
β-glucan	23.93 ± 1.51
Total glucan	27.32 ± 1.70

**Table 2:** Dietary compositions used in this study.

Ingredient (g/kg)	Experimental Group				
	ND	HFD + GEP [%]			
		0	1	3	5
Powdered GEP	0	0	10	30	50
Casein	200	200	200	200	200
Corn starch	457	260	250	230	210
Sucrose	200	200	200	200	200
Cellulose	50	50	50	50	50
Soybean oil	43	25	25	25	25
L-Cysteine	3	3	3	3	3
Choline bitartrate	2	2	2	2	2
Lard	0	215	215	215	215
Mineral mix	35	35	35	35	35
Vitamin mix	10	10	10	10	10
Total grams (g)	1000	1000	1000	1000	1000
Calories from fat (%)	10	45	45	45	45

ND: normal chow diet; HFD: high-fat diet; GEP: *Ganoderma lucidum* extract powder.

**Table 3:** qRT-PCR mouse primer sequences.

Gene	Forward Primer (5′ to 3′)	Reverse Primer (5′ to 3′)
FAS	TGCTCCAGGGATAACAGC	CCAAATCCAACATGGGACA
SCD1	ACCTGCCTCTTCGGGATTTT	GTCGGCGTGTGTTTCTGAGA
SREBP1c	AGCTGCGTGGTTTCCAACA	CCTCATGTAGGAATACCCTCCTCAT
18s rRNA	GTAACCCGTTGAACCCCATT	CCATCCAATCGGTAGTAGCG

FAS: fatty acid synthase, SCD1: stearoyl-CoA desaturase1, SREBP1c: sterol regulatory element-binding protein-1c. 18s rRNA: 18s ribosomal RNA.

**Table 4:** White adipose tissue (WAT) weight in HFD-induced obese mice treated with GEP.

Group	Subcutaneous WAT (g)	Epididymal WAT (g)	Mesenteric WAT (g)
ND	0.99 ± 0.05	0.8 ± 0.12	0.17 ± 0.05
HFD	3.4 ± 0.15	1.81 ± 0.04	0.62 ± 0.07
HFD + 1% GEP	3.07 ± 0.18	1.75 ± 0.04	0.43 ± 0.11
HFD + 3% GEP	2.77 ± 0.12	1.53 ± 0.06	0.17 ± 0.12
HFD + 5% GEP	2.29 ± 0.09	1.31 ± 0.11	0.06 ± 0.03

Data represent the mean ± SD of six to seven individual mice. Significant differences vs. the HFD group (control) were determined by one-way ANOVA; * *p* < 0.1, ** *p* < 0.01, *** *p* < 0.001. ND: normal chow diet group (control); HFD: high-fat diet group; HFD + GEP: high-fat diet and *Ganoderma lucidum* extract powder.

**Table 5:** Effects of *G. lucidum* extract on blood lipid and cholesterol levels.

Group.	TG (mg/dL)	TC (mg/dL)	HDL (mg/dL)	LDL (mg/dL)	FFA (mEq/dL)
ND	73.78 ± 1.5	110.72 ± 2.79	97.29 ± 3.83	35.92 ± 2.96	0.98 ± 0.04
HFD	123.04 ± 3.12	248.63 ± 8.19	160.65 ± 3.42	80.65 ± 3.00	1.98 ± 0.11
HFD + 1% GEP	118.91 ± 3.04	224.45 ± 6.83	150.14 ± 1.85	70.08 ± 1.83	1.93 ± 0.11
HFD + 3% GEP	104.09 ± 3.66	197.19 ± 8.61	139.23 ± 3.8	60.19 ± 3.71	1.66 ± 0.06
HFD + 5% GEP	88.83 ± 1.83	171.37 ± 9.62	119.38 ± 4.55	53.39 ± 3.74	1.39 ± 0.07

Data represent the mean ± SD of six to seven individual mice. Significant differences vs. the HFD group (control) were determined by one-way ANOVA; * *p* < 0.1, ** *p* < 0.01, *** *p* < 0.001. ND: normal chow diet group; HFD: high-fat diet group; HFD + GEP: high-fat diet and *Ganoderma lucidum* extract powder; TG: triglyceride; TC: total cholesterol; HDL: high-density lipoprotein-cholesterol; LDL: low-density lipoprotein-cholesterol; FFA: free fatty acid.

## Abbreviations

18s rRNA	18s ribosomal RNA
AAC	Area above the curve
ACC	Acetyl-CoA carboxylase
AKT1	AKT serine/threonine kinase 1
AMPK	5′ AMP-activated protein kinase
AS160	Akt substrate of 160 kDa
AUC	Area under the curve
C/EBPα	CCAAT-enhancer-binding protein α
CPT1	Carnitine palmitoyltransferase I
DAG	Diacylglycerol
FAS	Fatty acid synthase
FER	Feeding efficiency ratio
FFA	Free fatty acid
G6Pase	Glucose 6-phosphatase
GEP	*Ganoderma lucidum* extract powder
GLUT1	Glucose transporter type 1
GLUT4	Glucose transporter type 4
GTT	Glucose tolerance test
HDL	High-density lipoprotein-cholesterol
HFD	High-fat diet
HMG-CoA	β-Hydroxy β-methylglutaryl-CoA
IR	Insulin receptor
IRS1	Insulin receptor substrate 1
ITT	Insulin tolerance test
LDL	Low-density lipoprotein-cholesterol
mTOR	Mammalian target of rapamycin
ND	Normal diet
PEPCK	Phosphoenolpyruvate carboxykinase
PI3K	Phosphoinositide 3-kinase
PKC	Protein kinase C
PPARγ	Peroxisome proliferator-activated receptors γ
PTP1B	Protein tyrosine phosphatase 1B
S6K1	Ribosomal protein S6 kinase beta-1
SCD 1	Stearoyl-CoA desaturase 1
SD	Standard deviation
SREBP1c	Sterol regulatory element-binding protein-1c
TBC1D4	TBC1 Domain Family Member 4
TC	Total cholesterol
TG	Triglyceride
TORC2	mTOR Complex 2
WAT	White adipose tissue
